# No Gentleman Keeps a Bulldog

**Published:** 1890-10-18

**Authors:** W. Percy Laing

**Affiliations:** Brooklands, Romford


### Editor's Letter-Box.

[Our Correspondents are reminded that prolixity is a great bar to publication, and that brevity of style and conciseness of statement greatly facilitate early insertion.]

### "NO GENTLEMAN KEEPS A BULLDOG."

I have to thank you for kindly inserting my letter in your last issue. Your editorial note has, however, convinced me, more than ever, that the writer of the article to which it refers, knows absolutely nothing about the subject upon which he has written. I must really, therefore, ask you to give me space for one more word. First, he says, through you, that he has been an unwilling witness of more than one bulldog (?) fight, to which I reply that the fights which, I am sorry to say, do take place in all colliery districts are kept so secret, that only those in "the know" are able to attend. How then could he have been an unwilling witness? Secondly, I may tell him, sir, for his information, that the dogs used for fighting there are not bulldogs, but are a "terrier cross" (having bull blood in their veins certainly to give them endurance), and quite a different animal. This fact, sir, proves that he does not know a bulldog when he sees one, and therefore cannot be familiar with their ways. If, therefore, he does not know the dog and its ways, how can he possibly know the owners and theirs? I would ask him to read the two letters in the *Stockkeeper* of last Saturday, and, if they do not bring tears of shame into his eyes, they ought to. No, sir; let him confess at once that he has made a great mistake, and written upon a subject of which he is absolutely ignorant. Let him offer an apology to the "breed" and their owners, and, as the latter are usually as good tempered as their dogs, I think I can promise him forgiveness; at any rate, he will be forgiven by

